# Supplementary material for: Characterisation of the Effect of the Spatial Organisation of Hemicellulases on the Hydrolysis of Plant Biomass Polymer
Source: Int J Mol Sci. 2020 Jun 19;21(12):4360. doi: 10.3390/ijms21124360 (PMC7353053; doi:10.3390/ijms21124360)
Supplement: Supplementary file 1 [file ijms-21-04360-s001.pdf]

SUPPORTING INFORMATION FOR

Characterisation of the Effect of the Spatial Organisation of Hemicellulases on the Hydrolysis of Plant Biomass Polymer

Thomas Enjalbert [1], Marion De La Mare [2], Pierre Roblin [3], Louise Badruna [1], Thierry Vernet [4], Claire Dumon [1] and Cédric Y. Montanier [1,*]

[1] Toulouse Biotechnology Institute (TBI), Université de Toulouse, CNRS, INRAE, INSA, 31077 Toulouse, France; enjalber@insa-toulouse.fr (T.E.); louise.badruna@hotmail.fr (L.B.); cdumon@insa-toulouse.fr (C.D.)

[2] Toulouse White Biotechnology, UMS INRA 1337, UMS CNRS 3582, Institut National des Sciences Appliquées de Toulouse, 31077 Toulouse, France; mdelamare@enobraq.com

[3] Laboratoire de Génie Chimique, Université de Toulouse, CNRS, INPT, UPS, 31077 Toulouse, France; roblin@chimie.ups-tlse.fr

[4] Institut de Biologie Structurale, Univ., Grenoble Alpes, CEA, CNRS, IBS, F-38000 Grenoble, France; thierry.vernet@ibs.fr

* Correspondence: cedric.montanier@insa-toulouse.fr; Tel.: +33-(0)5-61-55-97-13

This supplementary Information contains 2 sections:

1. Figure S1 to S8
2. Tables S1 to S3

1. Figure S1 to S8

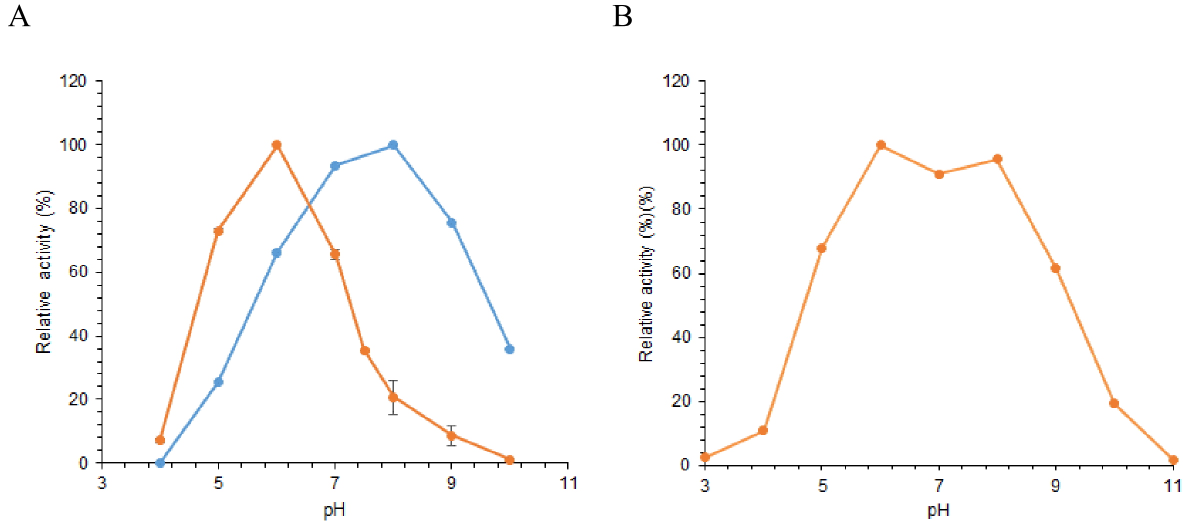

Figure S1. Effect of pH on activity of **A)** $_{\text{His}}$-In-*Np*Xyn11A (orange, from Montanier *et al*.[1]) and $_{\text{His}}$-Jo-*Bh*Xyl43 (blue, this work). Enzyme activity was measured in a pH range between 4 and 10 using 5 mM of 4-nitrophenyl-β-D-xylotrioside at 37 °C and 5 mM of 4-nitrophenyl-β-D-xylopyranoside at 45 °C, respectively. **B)** $_{\text{His}}$-In-*Np*Xyn11A. Enzyme activity was measured in a pH range between 3 and 11 using 2% Beech wood xylan mM at 37 °C.

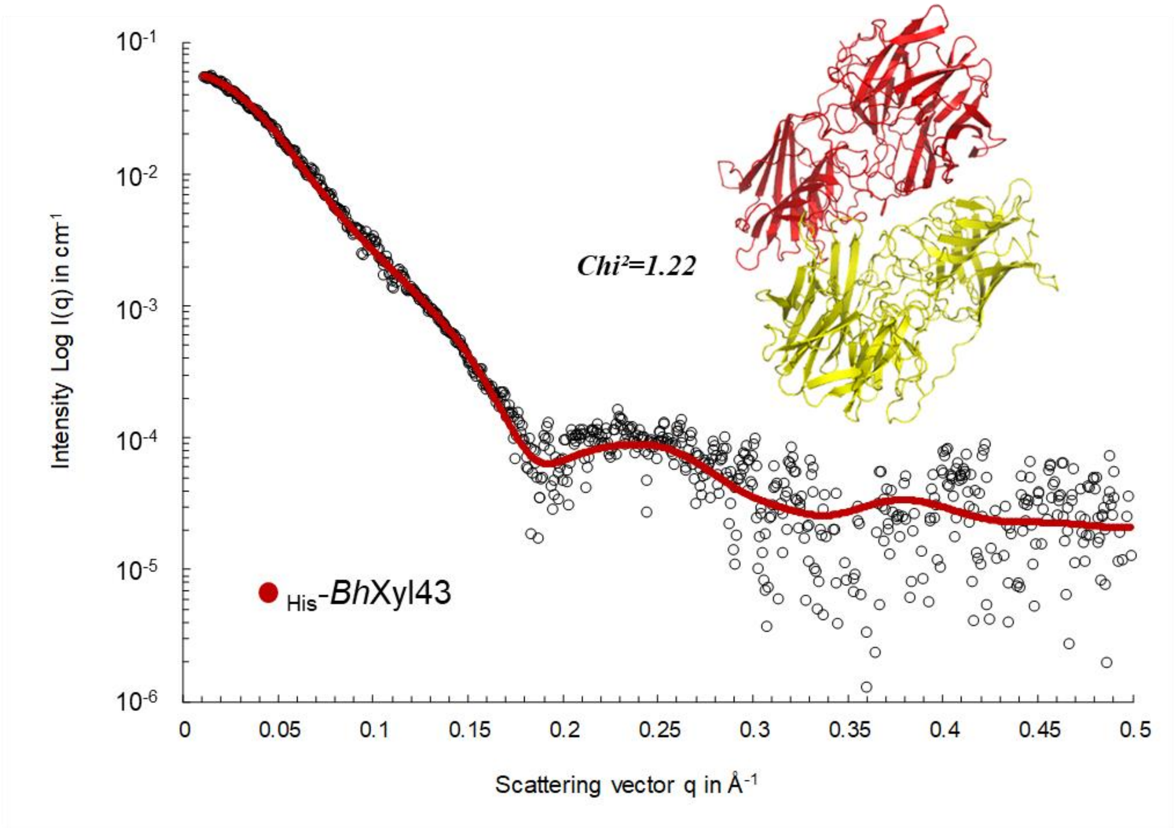

Figure S2. Superimposition of SAXS data from the beta-1,4-xylosidase $_{\text{His}}$-*Bh*Xyl43 plotted with dark dots and the SAXS curve computed from the crystallographic structure of the tetramer (PDB: 1YRZ) plotted in red line. The data are presented as a plot of Log I(q) vs. q with the intensity I(q) in cm-1 and scattering vector q in $Å^{-1}$.

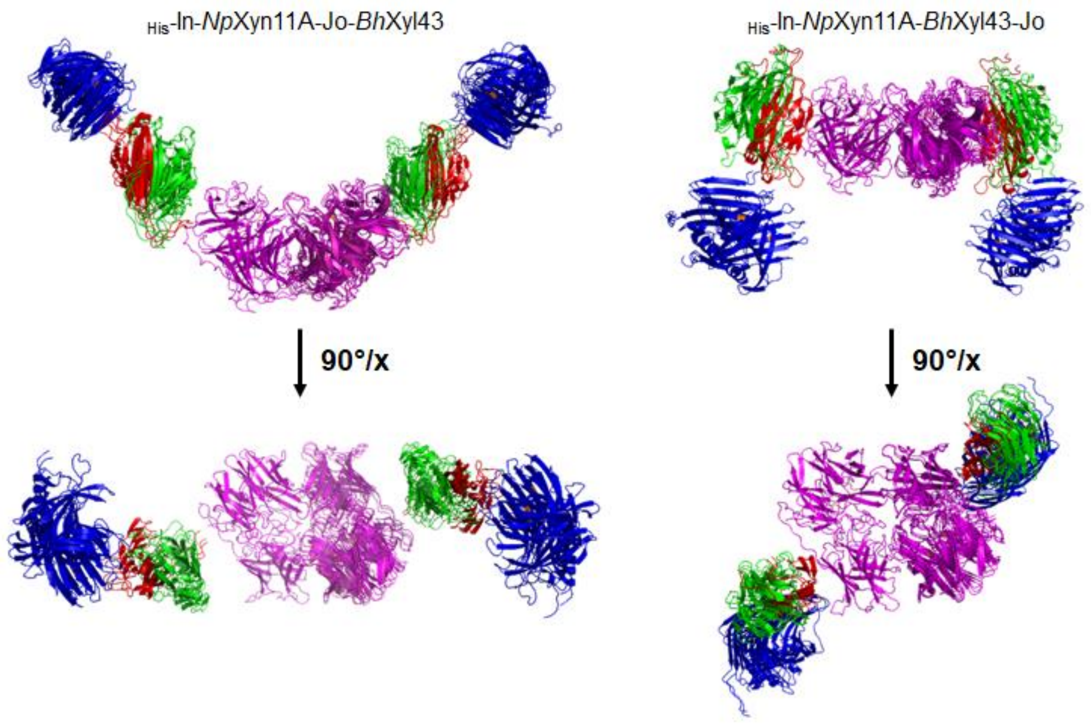

Figure S3. SAXS data compatible models of His-In-*Np*Xyn11A-Jo-*Bh*Xyl43 and His-In-*Np*Xyn11A-*Bh*Xyl43-Jo shown in two orientations. The three extreme structures from the set with a good fit are superimposed for both constructs. Domain *Np*Xyn11A is in blue, domain *Bh*Xyl43 is in magenta, domain In is in green and domain Jo is in red.

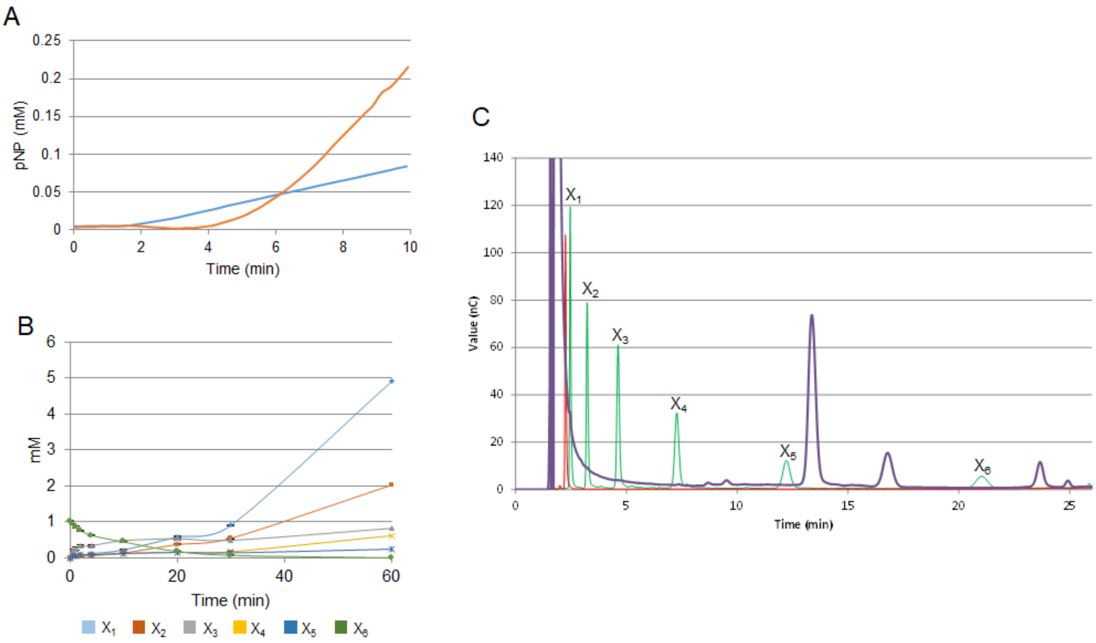

Figure S4. Activity of His-*Bh*Xyl43 on small oligosaccharides. (A) Activity of 8 nM of His-*Np*Xyn11A (blue) and His-*Bh*Xyl43 (red) over 5 mM of *p*NP-X_3 in 50 mM phosphate pH 7 supplemented with 1 mg/ml of BSA, at 37 °C, follow by the release of *p*NP at 401 nm. (B) HPAEC-PAD analysis of the hydrolysis of 1 mM xylohexaose with 60 nM of His-*Bh*Xyl43 in 50 mM Tris/HCl pH8 supplemented with 1 mg/ml of BSA, at 45 °C for 1 h. (C) HPAEC-PAD analysis of the hydrolysis of A^2XX by His-*Bh*Xyl43. Standard arabinose (red), standard xylooligosaccharides (green) and 1 mM A^2XX incubated with 60 nM of His-*Bh*Xyl43 in 50 mM Tris/HCl pH8 supplemented with 1 mg/ml of BSA, at 45 °C for 24 h (purple).

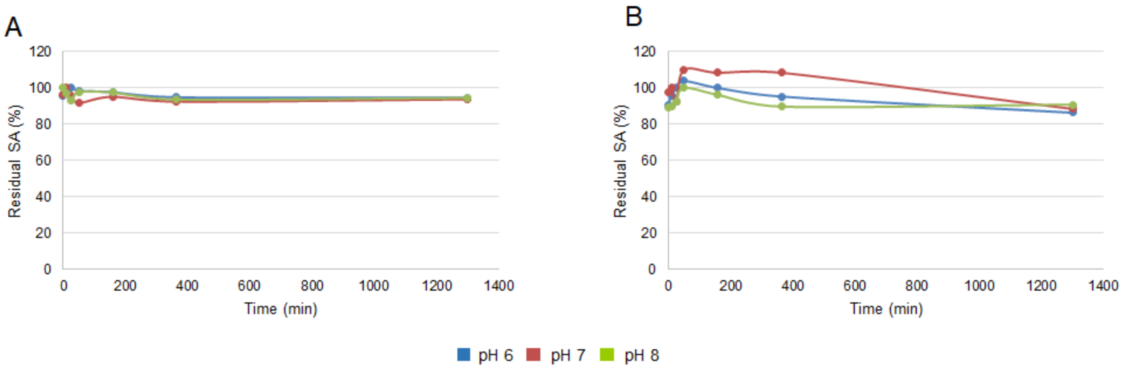

Figure S5. Enzyme stability in 50 mM Phosphate, supplemented with 1 mg/ml of BSA at 37 °C over 24 h, at pH 6, 7 and 8. (A) Residual specific activity of 5 nM of His-*Np*Xyn11A tested on 5 mM *p*NP-X$_3$. (B) Residual specific activity of 5 nM of His-*Bh*Xyl43 tested on 5 mM *p*NP-X.

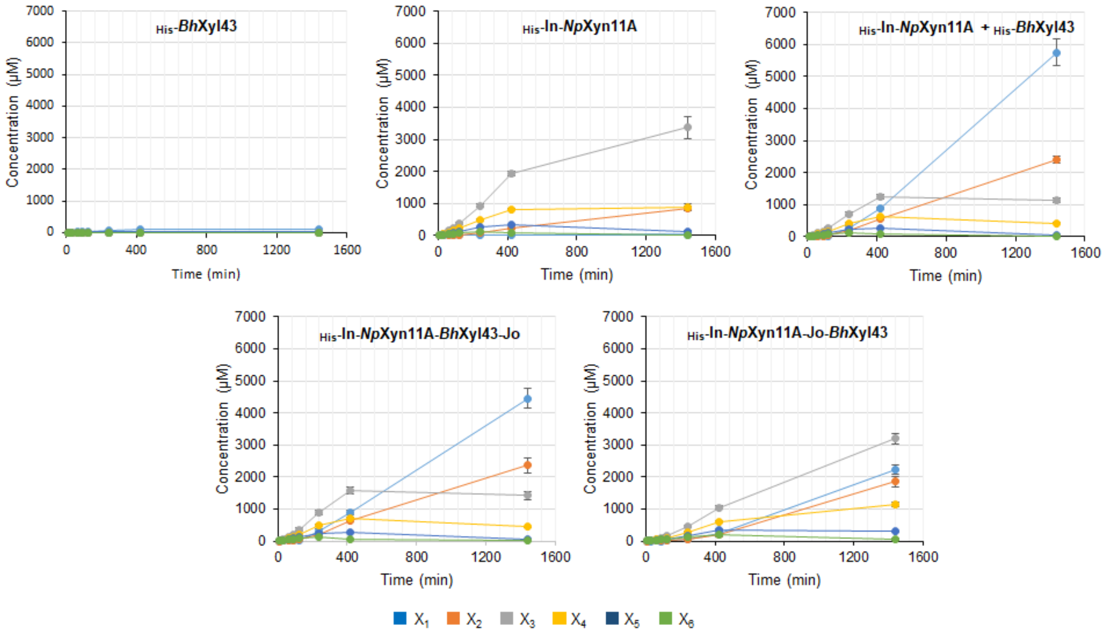

Figure S6. Example of curves of released oligosaccharides from X$_1$ to X$_6$ obtained from HPAEC-Pad analysis during 24 h of hydrolysis of 1% Beechwood xylan with various enzymes at pH 7 displaying the standard deviations. The average values were used to plot the graphs on Fig 5. Curved from His-*Bh*Xyl43 are presented as control.

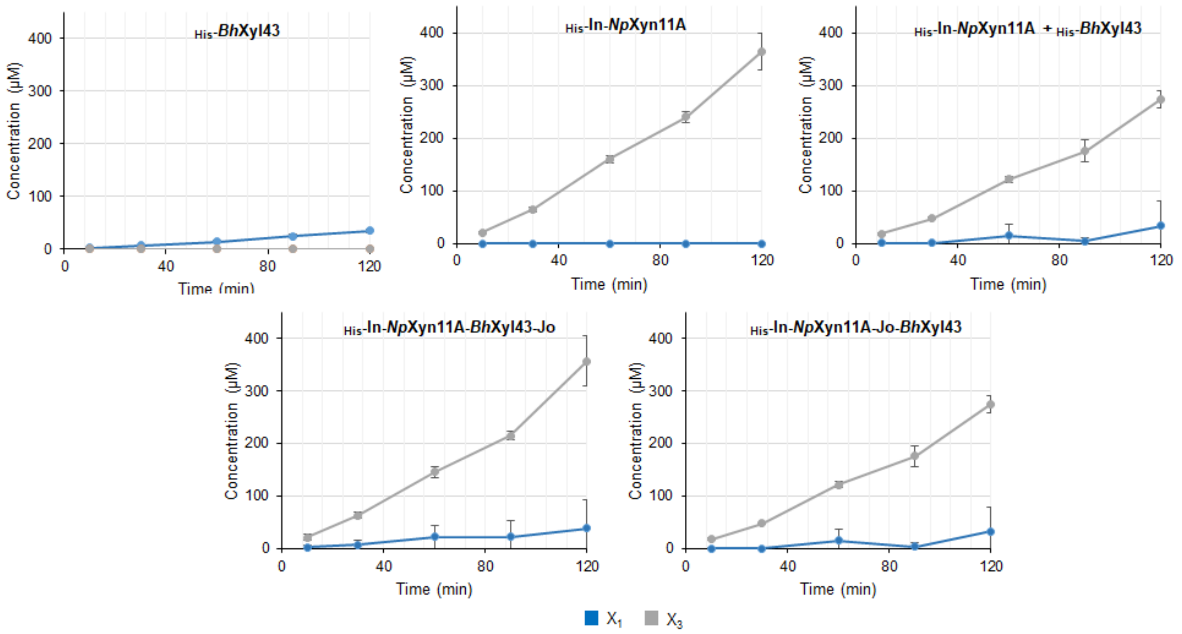

Figure S7. Closer view of the concentration of X_1 (blue line) and X_3 (gray line) released from 1% Beechwood xylan over the time (between 0 and 120 min). Data extracted from Fig 4 SI. Enzyme concentration set at 1 nM, 10 50 mM phosphate pH 7, 1 mg/ml BSA.

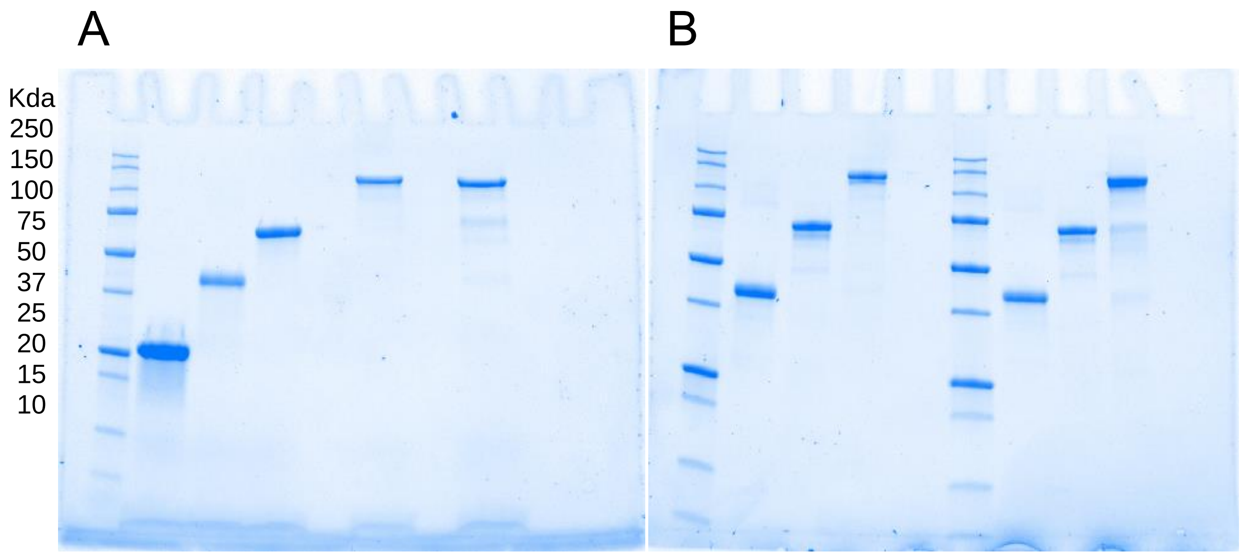

Figure 8. Original SDS-PAGE view. (**A**) Lanes: 1, molecular mass markers; 2, His-*Np*Xyn11A; 3, His-In-*Np*Xyn11A; 4, His-*Bh*Xyl43; 5, empty; 6, His-In-*Np*Xyn11A-Jo-*Bh*Xyl43; 7, empty; 8, His-In-*Np*Xyn11A-*Bh*Xyl43-Jo. (**B**) Lanes: 1, molecular mass markers; 2, His-In-*Np*Xyn11A; 3, His-Jo-*Bh*Xyl43; 4, His-In-*Np*Xyn11A-Jo-*Bh*Xyl43; 5, empty; 6, molecular mass markers; 7, His-In-*Np*Xyn11A; 8, His-Jo-*Bh*Xyl43; 9, His-In-*Np*Xyn11A-*Bh*Xyl43-Jo.

2. Tables S1 to S3

Table S1. : Biophysical parameters of $_{His}$-In-*Np*Xyn11A-Jo-*Bh*Xyl43 and $_{His}$-In-*Np*Xyn11A-*Bh*Xyl43-Jo complexes calculated from both SAXS curves shown in Figure S2 with ATSAS suite programs [2].

	R_g (Å)	D_{max} (Å)	Porod's volume (Å^3)	Calculated molecular weight (kDa)	Theorical molecular weight (kDa)
$_{His}$-*Bh*Xyl43	38.2	136	64,300	128.4	121.4
$_{His}$-In-*Np*Xyn11A-Jo-*Bh*Xyl43	56.4	230	278,000	189.1	223.9
$_{His}$-In-*Np*Xyn11A-*Bh*Xyl43-Jo	50.5	200	282,000	180.5	223.7

Table S2. Molar extinction coefficient and molecular weight of the enzymes studied in this work.

Enzyme	Molar extinction coefficient ($M^{-1}.cm^{-1}$)	Molecular weight (g/mol)
*Np*Xyn11A-$_{His}$	61,880	25,981
$_{His}$-In-*Np*Xyn11A	69,330	41,544
$_{His}$-*Bh*Xyl43	119,095	11,9095
$_{His}$-Jo-*Bh*Xyl43	129,525	72,164
Jo-*Bh*Xyl43	129,525	70,470
*Bh*Xyl43-Jo	129,525	70,339
$_{His}$-In-*Np*Xyn-Jo-*Bh*Xyl43	198,855	111,997
$_{His}$-In-*Np*Xyn-*Bh*Xyl43-Jo	198,855	111,886

Table S3. Synthetic gene sequences.

Jo-BhXyl43	***BhXyl43-Jo***
CCATGGGCGCTAGCCAGGATCCGTCTGACC	CCATGGGCGCTAGCGTCAATCGTATCCAAAAT
AGTATCCACAAACAGGGACTTATCCAGATG	CCTATTTTGCCAGGGTTTCATCCAGACCCATCC
TTCAAACACCTTATCAGATTATTAAGGTAG	ATTGTCCGTGTTGGTGATGATTACTATATCGCC
ATGGTTCGGAAAAAAACGGACAGCACAAG	ACCTCTACATTTGAATGGTTTCCTGGGGTGCGC
GCGTTGAATCCGAATCCATATGAACGTGTG	ATCCACCATTCTCGGGATTTAAAACATTGGCG
ATTCCAGAAGGTACACTTTCAAAGAGAATT	CTTTGTATCTAGTCCGCTGACCCGCACTTCCCA
TATCAAGTGAATAATTTGGATGATAACCAA	ACTAGACATGAAAGGGAATATGAACTCCGGCG
TATGGAATCGAATTGACGGTTAGTGGGAAA	GGATATGGGCGCCATGCCTAAGCTATCATGAC
ACAGTGTATGAACAAAAAGATAACGTCGAC	GGAACCTTTTATTTGATCTATACTGATGTGAAG
ATGGTCAATCGTATCCAAAATCCTATTTTGC	CAATGGCACGGTGCCTTCAAAGACGCGCACAA
CAGGGTTTCATCCAGACCCATCCATTGTCC	CTATTTAGTGACGGCACAAAACATTGAAGGGC
GTGTTGGTGATGATTACTATATCGCCACCTC	CGTGGTCGGACCCGATTTACTTAAACAGTAGC
TACATTTGAATGGTTTCCTGGGGTGCGCATC	GGCTTTGACCCGTCCCTGTTTCACGATGACGAT
CACCATTCTCGGGATTTAAAACATTGGCGC	GGCCGAAAATGGCTCGTTAACATGATCTGGGA
TTTGTATCTAGTCCGCTGACCCGCACTTCCC	CTACCGCAAAGGAAACCATCCTTTTGCCGGAA
AACTAGACATGAAAGGGAATATGAACTCCG	TTATTTTGCAAGAATACTCAGAAGCAGAACAA
GCGGGATATGGGCGCCATGCCTAAGCTATC	AAACTTGTCGGGCCTGTGAAAAATATCTATAA
ATGACGGAACCTTTTATTTGATCTATACTGA	AGGGACCGACATTCAGCTAACAGAGGGACCGC
TGTGAAGCAATGGCACGGTGCCTTCAAAGA	ACCTCTATAAGAAAGATGGTTATTATTATTTAC

CGCGCACAACTATTTAGTGACGGCACAAAA	TTGTTGCCGAAGGAGGGACGGAATACGAACAC
CATTGAAGGGCCGTGGTCGGACCCGATTTA	GCCGCGACCCTCGCCCGCTCACAGTCAATTGA
CTTAAACAGTAGCGGCTTTGACCCGTCCCT	CGGACCCTATGAGACCGACCCGAGTTATCCAC
GTTTCACGATGACGATGGCCGAAAATGGCT	TCGTCACATCGACTGGCCAGCCGGAATTGGCG
CGTTAACATGATCTGGGACTACCGCAAAGG	TTGCAAAAGGCCGGACACGGTAGCCTCGTAGA
AAACCATCCTTTTGCCGGAATTATTTTGCAA	AACCCAGAACGGCGAATGGTATCTCGCTCACT
GAATACTCAGAAGCAGAACAAAAACTTGTC	TGTGCGGTCGCCCATTAAAAGGAAAGTACTGC
GGGCCTGTGAAAAATATCTATAAAGGGACC	ACACTCGGCAGGGAAACAGCCATTCAAAAAGT
GACATTCAGCTAACAGAGGGACCGCACCTC	AAACTGGACCGAGGATGGCTGGCTGCGCATCG
TATAAGAAAGATGGTTATTATTATTTACTTG	AGGATGGCGGCAATCACCCGTTGCGTGAAGTG
TTGCCGAAGGAGGGACGGAATACGAACAC	ACGGCACCTGACCTTCCAGAGCACCCATTCGA
GCCGCGACCCTCGCCCGCTCACAGTCAATT	AAAAGAACCCGAGCTCGATGATTTTGACGCAC
GACGGACCCTATGAGACCGACCCGAGTTAT	CCCAGCTGCACCATCAATGGAACACGCTGCGC
CCACTCGTCACATCGACTGGCCAGCCGGAA	ATCCCTGCCGACCCATCATGGTGCTCGCTCGA
TTGGCGTTGCAAAAGGCCGGACACGGTAGC	GGAACGTCCGGGCCATTTACGACTGCGCGGGA
CTCGTAGAAACCCAGAACGGCGAATGGTAT	TGGAGTCCCTCACTTCCGTCCACTCGCAAAGTT
CTCGCTCACTTGTGCGGTCGCCCATTAAAA	TAGTCGCCCGCAGGCAGCAGTCCTTCCACTGC
GGAAAGTACTGCACACTCGGCAGGGAAAC	GAAGTTGAGACAAAGCTAGAGTATCAGCCAGA
AGCCATTCAAAAAGTAAACTGGACCGAGG	ATCGTTTCAACATATGGCTGGGCTTGTCATTTA
ATGGCTGGCTGCGCATCGAGGATGGCGGCA	CTATGACACAGAAGATCATGTCTATTTGCACG
ATCACCCGTTGCGTGAAGTGACGGCACCTG	TAACCTGGCACGAGGAAAAGGGTAAATGTCTA
ACCTTCCAGAGCACCCATTCGAAAAAGAAC	CAAATCATACAGACAAAGGGCGGAAACTATG
CCGAGCTCGATGATTTTGACGCACCCCAGC	ACGAATTGCTTGCGTCACCGATCCCACTGGCA
TGCACCATCAATGGAACACGCTGCGCATCC	GAAGAAAAGGCGGTTTATTTGAAGGGGCGCAT
CTGCCGACCCATCATGGTGCTCGCTCGAGG	TCACCGTGAAACGATGCACCTCTATTTCAAAC
AACGTCCGGGCCATTTACGACTGCGCGGGA	AAGAGGGAGAAGCGGAATGGCAGCCTGTGGG
TGGAGTCCCTCACTTCCGTCCACTCGCAAA	GCCAACGATTGATGTGACCCACATGTCCGACG
GTTTAGTCGCCCGCAGGCAGCAGTCCTTCC	ATTCAGCGAAGCAAGTTCGATTTACCGGCACA
ACTGCGAAGTTGAGACAAAGCTAGAGTATC	TTTGTCGGCATGGCTACGCAAGACTTGAGCGG
AGCCAGAATCGTTTCAACATATGGCTGGGC	AACGAAAAAGCCAGCCGATTTTGATTACTTTC
TTGTCATTTACTATGACACAGAAGATCATG	GCTATAAAGAACTAGATCAACAGGATCCGTCT
TCTATTTGCACGTAACCTGGCACGAGGAAA	GACCAGTATCCACAAACAGGGACTTATCCAGA
AGGGTAAATGTCTACAAATCATACAGACAA	TGTTCAAACACCTTATCAGATTATTAAGGTAG
AGGGCGGAAACTATGACGAATTGCTTGCGT	ATGGTTCGGAAAAAAACGGACAGCACAAGGC
CACCGATCCCACTGGCAGAAGAAAAGGCG	GTTGAATCCGAATCCATATGAACGTGTGATTC
GTTTATTTGAAGGGGCGCATTCACCGTGAA	CAGAAGGTACACTTTCAAAGAGAATTTATCAA
ACGATGCACCTCTATTTCAAACAAGAGGGA	GTGAATAATTTGGATGATAACCAATATGGAAT
GAAGCGGAATGGCAGCCTGTGGGGCCAAC	CGAATTGACGGTTAGTGGGAAAACAGTGTATG
GATTGATGTGACCCACATGTCCGACGATTC	AACAAAAAGATAACGTCGACTAACAAGCTT
AGCGAAGCAAGTTCGATTTACCGGCACATT	
TGTCGGCATGGCTACGCAAGACTTGAGCGG	
AACGAAAAAGCCAGCCGATTTTGATTACTT	
TCGCTATAAAGAACTAGATCAATAACAAGC	
TT	

Reference

1. Montanier, C.Y.; Fanuel, M.; Rogniaux, H.; Ropartz, D.; Di Guilmi, A.-M.; Bouchoux, A. Changing surface grafting density has an effect on the activity of immobilized xylanase towards natural polysaccharides. *Sci. Rep.* **2019**, *9*, 5763, doi:10.1038/s41598-019-42206-w.
2. Konarev, P.V.; Volkov, V.V.; Sokolova, A.V.; Koch, M.H.J.; Svergun, D.I. PRIMUS : A Windows PC-based system for small-angle scattering data analysis. *J. Appl. Crystallogr.* **2003**, *36*, 1277–1282, doi:10.1107/S0021889803012779.

.
